# Advanced diffusion imaging for assessing normal white matter development in neonates and characterizing aberrant development in congenital heart disease

**DOI:** 10.1016/j.nicl.2018.04.032

**Published:** 2018-05-01

**Authors:** S. Karmacharya, B. Gagoski, L. Ning, R. Vyas, H.H. Cheng, J. Soul, J.W. Newberger, M.E. Shenton, Y. Rathi, P.E. Grant

**Affiliations:** a Brigham and Women's Hospital, Harvard Medical School, Boston, MA, United States; b Boston Children's Hospital, Harvard Medical School, Boston, MA, United States; c Boston VA Healthcare, Boston, MA, United States

**Keywords:** Neonatal white matter development, Diffusion MRI, Congenital heart disease

## Abstract

**Background:**

Elucidating developmental trajectories of white matter (WM) microstructure is critically important for understanding normal development and regional vulnerabilities in several brain disorders. Diffusion Weighted Imaging (DWI) is currently the method of choice for in-vivo white matter assessment. A majority of neonatal studies use the standard Diffusion Tensor Imaging (DTI) model although more advanced models such as the Neurite Orientation Dispersion and Density Imaging (NODDI) model and the Gaussian Mixture Model (GMM) have been used in adult population. In this study, we compare the ability of these three diffusion models to detect regional white matter maturation in typically developing control (TDC) neonates and regional abnormalities in neonates with congenital heart disease (CHD).

**Methods:**

Multiple b-value diffusion Magnetic Resonance Imaging (dMRI) data were acquired from TDC neonates (N = 16) at 38 to 47 gestational weeks (GW) and CHD neonates (N = 19) aged 37 weeks to 41 weeks. Measures calculated from the diffusion signal included not only Mean Diffusivity (MD) and Fractional Anisotropy (FA) derived from the standard DTI model, but also three advanced diffusion measures, namely, the fiber Orientation Dispersion Index (ODI), the isotropic volume fraction (V_iso_), and the intracellular volume fraction (V_ic_) derived from the NODDI model. Further, we used two novel measures from a non-parametric GMM, namely the Return-to-Origin Probability (RTOP) and Return-to-Axis Probability (RTAP), which are sensitive to axonal/cellular volume and density respectively. Using atlas-based registration, 22 white matter regions (6 projection, 4 association, and 1 callosal pathways bilaterally in each hemisphere) were selected and the mean value of all 7 measures were calculated in each region. These values were used as dependent variables, with GW as the independent variable in a linear regression model. Finally, we compared CHD and TDC groups on these measures in each ROI after removing age-related trends from both the groups.

**Results:**

Linear analysis in the TDC population revealed significant correlations with GW (age) in 12 projection pathways for MD, V_ic_, RTAP, and 11 pathways for RTOP. Several association pathways were also significantly correlated with GW for MD, V_ic_, RTAP, and RTOP. The right callosal pathway was significantly correlated with GW for V_ic_. Consistent with the pathophysiology of altered development in CHD, diffusion measures demonstrated differences in the association pathways involved in language systems, namely the Uncinate Fasciculus (UF), the Inferior Fronto-occipital Fasciculus (IFOF), and the Superior Longitudinal Fasciculus (SLF). Overall, the group comparison between CHD and TDC revealed lower FA, V_ic,_ RTAP, and RTOP for CHD bilaterally in the a) UF, b) Corpus Callosum (CC), and c) Superior Fronto-Occipital Fasciculus (SFOF). Moreover, FA was lower for CHD in the a) left SLF, b) bilateral Anterior Corona Radiata (ACR) and left Retrolenticular part of the Internal Capsule (RIC). V_ic_ was also lower for CHD in the left Posterior Limb of the Internal Capsule (PLIC). ODI was higher for CHD in the left CC. RTAP was lower for CHD in the left IFOF, while RTOP was lower in CHD in the: a) left ACR, b) left IFOF and c) right Anterior Limb of the Internal Capsule (ALIC).

**Conclusion:**

In this study, all three methods revealed the expected changes in the WM regions during the early postnatal weeks; however, GMM outperformed DTI and NODDI as it showed significantly larger effect sizes while detecting differences between the TDC and CHD neonates. Future studies based on a larger sample are needed to confirm these results and to explore clinical correlates.

## Highlights

• Determine developmental trajectories of several white matter regions in typically developing neonates.
• Compare white matter abnormalities in brain regions between congenital heart disease neonates and typically developing controls.
• Compare DTI, NODDI and Gaussian mixture model (GMM) and found GMM to be most sensitive to tissue abnormalities.

## 1 Introduction

In the young adult human brain, white matter (WM) contains 149,000 to 176,000 km of myelinated axons (Marner et al., 2003). WM is the wiring structure of the brain that allows effective communication between different cortical gray matter areas that have distinct functional characteristics. The organization and maturation of axonal pathways in regional WM structures follow heterogeneous trajectories beginning in the prenatal to early postnatal stages. For example, post-mortem studies have shown that the Posterior Limb of Internal Capsule (PLIC) has partial myelination seen at birth (Brody et al., 1987). On the other hand, in the Corpus Callosum (CC), myelination progresses much later during the postnatal period (Kinney et al., 1988). Further, the Superior Longitudinal Fascicle (SLF), an association pathway, has a slow maturation period that extends into adulthood (Zhang et al., 2007). Origins of several neurodevelopmental and psychiatric disorders have been associated with the dysregulation in the organization and maturation of the WM regions (Dietrich et al., 1988; Tkachev et al., 2003; Pujol et al., 2004; Cascio et al., 2006; Courchesne et al., 2007; Brennand et al., 2011).

Similarly, individuals with Congenital Heart Disease (CHD) make up a population that has been associated with a history of neurologic and neuro-developmental impairments such as motor and visuospatial skills, as well as cognitive impairments such as working memory, attention, and language (Bellinger et al., 1999; Limperopoulos et al., 1999; Hovels-Gurich, 2002; Bellinger et al., 2003a; Bellinger et al., 2003b; Hövels-Gürich et al., 2007, Hövels-Gürich et al., 2008). Moreover, previous neonatal brain imaging studies have shown evidence of delayed development of pyramidal tracts, presence of white matter injury associated with hypoxia, lower gray and white matter volume, abnormal cerebral blood flow, and abnormal metabolism in CHD neonates (Mahle et al., 2002; Licht et al., 2004; Pas te et al., 2005; Partridge et al., 2006; McQuillen et al., 2007; Petit et al., 2009; Ortinau et al., 2012; Dimitropoulos et al., 2013; Dehaes et al., 2015). Given the importance of the WM during development and its association with neurodevelopmental disorders, it is crucial to accurately characterize the normal development in typically developing neonates and potentially to detect aberrant development of regional WM structures in neonates with CHD. Increasing our understanding about affected WM regions will allow for better characterization of WM abnormalities which are needed to guide the search for potential etiologies and to understand cognitive outcomes. Advanced MRI techniques such as diffusion Magnetic Resonance Imaging (dMRI) have been crucial in characterizing the early organization of regional WM in neonates, and are essential for investigating abnormalities in early WM development. Moreover, past studies have used measures such as Diffusion Tensor Imaging (DTI) and Neurite Orientation Dispersion and Density Imaging (NODDI) to study the normal development and to explore WM abnormalities in CHD (Kunz et al., 2014).

While DTI (Basser et al., 1994) is a classic method used to determine WM structural integrity, it has several limitations: 1) it assumes the existence of only a single fiber population oriented in one particular direction (Tuch et al., 2002), and 2) it assumes Gaussian diffusion with no restriction to motion of water molecules, which is an oversimplification of the underlying biological process. While FA derived from DTI has been reported to be sensitive to myelination of axons (Chenevert et al., 1990), it is not specific to one particular type of abnormality, i.e., several different biological processes can produce similar types of alterations in FA. Hence, it cannot provide specific information about the nature of the microstructural abnormalities (Westin et al., 2016).

To overcome the limitations of DTI, several compartmental models of diffusion have been proposed. For example, the NODDI (Zhang et al., 2012) model attempts to characterize neural tissue structure with three different compartments representing restricted, hindered, and isotropic diffusion. In addition, NODDI models the dispersion of axonal fibers with the use of an Orientation Dispersion Index (ODI). One key limitation of the NODDI model is that it requires an a-priori chosen value to be set for the diffusivity along the axons, which may not be accurate in all regions of the developing neonate brain and may be difficult to estimate when development or disease alters brain structure (Lampinen et al., 2017). Nevertheless, NODDI has been used to study the microstructure of the neonate brain (Kunz et al., 2014). However, the potential of this model to characterize better regional WM tract development in normal neonates and to detect changes in neonates with CHD remains unexplored.

An alternative approach to DTI and NODDI is the Gaussian Mixture Model (GMM). Unlike DTI, the GMM does not assume Gaussian diffusion and, unlike NODDI, the GMM is independent of tissue models, i.e., it does not make any assumption about the number of compartments or their diffusivities. Instead, the GMM estimates the Ensemble Average diffusion Propagator (EAP), which describes the probability distribution of the displacement of water molecules within an experimentally set diffusion time (Cheng, 2012; Özarslan et al., 2013; Ning et al., 2015). The EAP allows calculation of several tissue microstructure related scalar measures, such as the Return-to-Origin Probability (RTOP) and Return-to-Axis Probability (RTAP) (Ning et al., 2015). RTOP is the probability that a water molecule returns to its starting position (the origin) within the experimental diffusion time. Thus, in a highly restricted or hindered medium, we expect high RTOP values, whereas, in the case of free diffusion, the RTOP values will be low, as there is very little probability that the water molecules will return to their starting position. It has also has been reported in the physics literature that RTOP is inversely proportional to the volume filled by air in a porous material (Özarslan et al., 2013; Ning et al., 2015). In the case of complex biological brain tissue, it provides a measure of the total volume within which water diffusion occurs. This, in turn, is related to the cellular and axonal volume, size, and myelination. For instance, a densely packed set of myelinated axons will leave very little extra-cellular space within which water diffuses (since most of the space is occupied by myelin and cell membranes), leading to small diffusion volume but higher RTOP (since volume is inversely proportional). Additionally, one can compute RTAP, which measures the probability of water molecules returning back to the axis or line representing the principal diffusion direction. RTAP is inversely proportional to the transverse cross-sectional area of the fiber bundles and hence is related to axon diameter, packing and amount of myelination. Since the GMM assumes very little about the diffusion process, it can be very sensitive to abnormalities in the diffusion process but lacks the ability to provide model specific information such as fiber orientation dispersion. The potential for GMM to detect and to characterize white matter abnormalities in CHD is further explored in this work.

In this paper, we explore the potential of both NODDI and the GMM to characterize regional WM development in TDC neonates as well as to detect abnormalities in specific WM regions in neonates with CHD. We also compare these novel measures with the standard DTI based measure of fractional anisotropy (FA) and mean diffusivity (MD). Thus, the present study also aims to compare different dMRI models to determine: 1) the sensitivity of different diffusion measures in their ability to detect age-related (cross-sectional) regional WM changes in TDC neonates, and 2) which measures detect regional WM differences in CHD compared to TDC neonates.

## 2 Materials and methods

The cross-sectional study included 16 Typically Developing Controls (TDC) (mean age: 42.03 ± 2.28 weeks; Gender: 12 females, 4 males) and 19 with Congenital Heart Disease (CHD) (mean age: 39.54 ± 1.08 weeks; Gender: 3 females, 16 males). All the CHD and TDC neonates were screened at the Boston Children's Hospital and have birth gestational week (GW) greater than 36 weeks. The TDC cohort included typically developing neonates recruited from a well baby nursery with uneventful deliveries, including Apgar scores greater than 8 at 5 minutes and no clinical signs or symptoms concerning any brain disorder. The CHD cohort included three different subtypes of CHD: 6 neonates with transposition of great arteries (TGA), 10 single ventricle physiology, and 3 bi-ventricle physiology. The details of the specific cardiac anomalies are summarized in Table 1. In addition, injuries observed in T1, T2, diffusion and Susceptibility Weighted Images are summarized in Table 2. MR acquisition was performed on a 3 T TimTrio Siemens system using a 32-channel head coil and a simultaneous multi-slice acquisition sequence (Setsompop et al., 2012). A multi-b-value DWI protocol was used with a total of 81 axial slices acquired with four non-diffusion weighted images (at b = 0 s/mm^2^). Acquisition parameters were: TR = 3700 ms, TE = 104 ms, flip angle = 90°, 2 mm isotropic spatial resolution, with two b-value shells at b = {1000, 2000} s/mm^2^ each shell having 30 gradient directions, with a total acquisition time of about 6 min.

**Table 1:** Details of cardiac anomaly including specific diagnosis, group and obstruction in congenital heart disease neonates (N = 19).

	Diagnosis for each of the 19 CHD neonates	Group	Obstruction
CHD 1	dTGA (dextro-Transposition of the Great Arteries)	TGA	
CHD 2	HLHS (Hypoplastic Left Heart Syndrome) variant, MA (Mitral Atresia), VSD (Ventricular Septal Defect)	SingleV	Left-sided
CHD 3	Hypoplastic TV/RV (Tricuspid Valve/Right Ventricle), dTGA, VSD	SingleV	Right-sided
CHD 4	HLHS	SingleV	Left-sided
CHD 5	CoA (Coarctation of the Aorta), PDA (Patent Ductus Arteriosus), PFO (Patent Foramen Ovale), VSD	Bi-V	
CHD 6	Aortic Arch Hypoplasia, VSD	Bi-V	
CHD 7	HLHS (MS/AS (Mitral Stenosis/Aortic St enosis))	SingleV	Left-sided
CHD 8	PA (Pulmonary Atresia), L-TGA, Dextrocardia	SingleV	Right-sided
CHD 9	dTGA	TGA	
CHD 10	Type B IAA (Interrupted Aortic Arch), VSD	Bi-V	
CHD 11	Tricuspid Atresia Type IB	SingleV	Right-sided
CHD 12	HLHS	SingleV	Left-sided
CHD 13	Pulmonary Atresia	SingleV	Right-sided
CHD 14	dTGA	TGA	
CHD 15	dTGA/IVS (Intact Ventricular Septum)	TGA	
CHD 16	dTGA, ASD (Atrial Septal Defect), VSD, Hypoplastic Arch	TGA	
CHD 17	HLHS	SingleV	Left-sided
CHD 18	DILV (Double Inlet Left Ventricle) with large VSD	SingleV	Right-sided
CHD 19	TGA/VSD	TGA

**Table 2:** Imaging abnormalities evident on T1, T2, diffusion and Susceptibility Weighted Images in congenital heart disease neonates (N = 19). * = obtained post operatively; R = right; L = left; CC = corpus callosum; WM = white matter; IVH = intraventricular hemorrhage; ↓, = decreased; ↑ = increased; NC = no change. Note subjects 14, 16 and 18 were imaged post operatively and the remaining 16 preoperatively.

	WM Injury in Tl and T2	Diffusion (MD and FA)	Hemorrhage in SWI
CHD 1	1 punctate Tl foci, 1 T2 bright focus	–	Multiple punctate cerebellar
CHD 2	1 punctate Tl foci	–	–
CHD 3	9 punctate Tl foci	–	–
CHD 4	6 punctate Tl foci	L Peritrigonal WM Splenium of CC (↓, ↓)	–
CHD 5	–	–	–
CHD 6	–	–	–
CHD 7	1 punctate Tl foci	–	–
CHD 8	–	Small R Occipital focus (↓, ↑)	–
CHD 9	–	–	2 punctate cerebellar
CHD 10	–	–	–
CHD 11	–	–	IVH
CHD 12	1 punctate Tl foci	–	–
CHD 13	–	–	–
CHD 14	19 punctate Tl foci	L Peritrigonal WM (↓, NC)	–
CHD 15	–	–	–
CHD 16	8 punctate Tl foci	–	2 punctate WM
CHD 17	–	–	Multiple punctate cerebellar
CHD 18	–	Small L WM focus (↓, ↓)	–
CHD 19	–	–	Caudothalic notch, IVH

### 2.1 Data preprocessing

All DWI images first went through a semi-automatic quality control pipeline (in-house MATLAB script) that detects signal intensity drop in each slice by measuring the divergence of signal intensity between adjacent slices. Any dMRI volume with significant motion artifact or signal loss was manually inspected and removed. Next, we performed head motion and eddy current correction for each data set using the FSL FLIRT software (Jenkinson and Smith, 2001; Jenkinson et al., 2002) where the gradient directions were appropriately corrected using the rotation parameters obtained from rigid registration to the b = 0 image.

### 2.2 Diffusion Tensor Imaging Model

Diffusion tensors were estimated at each voxel in Slicer Version 4 (http://www.slicer.org), using weighted linear least squares fitting to obtain the eigenvectors and eigenvalues, which were then used to calculate Fractional Anisotropy (FA) and Mean Diffusivity (MD).

### 2.3 Neurite Orientation Dispersion and Density Imaging (NODDI)

The NODDI model decomposes the dMRI signal into three subdivisions or compartments: the intra-cellular signal, the extra-cellular signal, and the isotropic compartment (Zhang et al., 2012). The model incorporates the dispersion of fibers while modeling the intra-cellular compartment using a Watson distribution function, which can be used to estimate the fiber ODI (Zhang et al., 2012). The intra-cellular compartment (V_ic_) of the NODDI model represents diffusion within the axons and cells. The isotropic compartment (V_iso_) estimates the volume fraction of freely diffusing extracellular water such as cerebrospinal fluid. The normalized signal, A, in the NODDI model is given by the following equation:

(A.1) $$A=\left(1-V_{\mathrm{iso}}\right)\left(V_{\mathrm{ic}}A_{\mathrm{ic}}+\left(1-V_{\mathrm{ic}}\right)A_{\mathrm{ec}}\right)+V_{\mathrm{iso}}A_{\mathrm{iso}}$$

where, *A*_*ic*_ is the signal contribution from the intra-cellular compartment and *A*_*ec*_ is the signal due to diffusion in the extra-cellular space. We should note that the NODDI model assumes an a-priori fixed diffusion coefficient, 1.7 × 10^−3^ mm^2^/ s, along the principal diffusion direction for both the intra- and extra-cellular compartments. We used the default fixed parameters while estimating all the measures (Orientation Dispersion Index, Intra-cellular Volume Fraction, and Isotropic Volume Fraction) for the NODDI model using the MATLAB software that is publicly available (Zhang et al., 2012).

### 2.4 Gaussian Mixture Model

The GMM estimates the 3-dimensional probability distribution of the displacement of water molecules in a given experimental time. Analytical expressions for computing measures such as RTOP and RTAP were proposed in Ning et al. (2015), which we used in this work. Unlike compartmental models, this method utilizes a directional radial basis function for reconstructing the diffusion signal as a linear combination of Gaussian basis functions centered across q-space, and not just at the origin. As noted previously, these measures are sensitive to the axonal and cellular size, density, and volume. All of these measures were computed using the publicly available software (https://github.com/LipengNing/RBF-Propagator/).

### 2.5 Atlas registration

To analyze the microstructure in different brain regions, we used the John Hopkins Neonate Atlas (JHNA) (Oishi et al., 2011) to define the regions of interests (ROIs) in the neonate brains. This atlas contains 122 WM and gray matter ROIs, which have been used to study brain development in neonates with an age range of 37 to 53 GW (Oishi et al., 2011). All diffusion measures were computed in the native subject space, and were up-sampled linearly to the voxel size of 0.6 mm^3^ to match the resolution of JHNA. Advanced Normalization Tools (ANTs) (Avants et al., 2008) were used to first affine transform the JHU-neonate FA atlas to each neonate subject FA space, followed by a non-rigid registration. The registration matrices obtained during these transformations were applied to the JHU-neonate label map to obtain subject-specific definitions of several WM regions. In this work, we focused our analysis on deep WM regions because deformable atlas registration was robust and consistent in registering the JHU-neonate label primarily in the deep WM regions. Mean MD, FA, V_ic_, V_iso,_ ODI, RTAP, RTOP were calculated from 22 ROIs (11 left hemispheres and 11 right hemispheres) defined by the JHU atlas for each individual subject.

### 2.6 Regional Developmental Trajectories in typically developing controls (TDC)

Our first analysis focused on understanding the changes in diffusion measures in TDC neonates at different postnatal weeks (age) in all the 11 WM regions. We grouped the 11 ROIs into two groups: 1) projection fibers, and 2) association and callosal fibers (see Fig. 2). Regression analysis with respect to GW was performed for MD, FA, V_ic_, V_iso,_ ODI, RTAP and RTOP for the TDCs using R software package. False discovery rate (FDR) correction was used to account for multiple comparisons. We particularly focused on three WM regions namely, PLIC, CC and SLF to perform qualitative comparison to determine which ROI has the highest and lowest values during the early postnatal developmental stage.

### 2.7 WM abnormalities in CHD

Since there were age differences between the CHD and TDC neonates, we first regressed out the effect of age from all TDC and CHD subjects. Subsequently, a *t*-test was used to estimate statistical differences between the two groups for each of the 7 measures and the 22 WM regions (a total of 154 *t*-tests). FDR correction was used to account for multiple hypothesis testing.

## 3 Results

### 3.1 Developmental trajectories in typically developing controls (TDC)

Linear regression model allows estimating the maturation of WM regions as a function of age. Age-related changes in diffusion measures in the 22 WM (11 LH and 11 RH) regions included: Uncinate Fasciculus (UF), Inferior Fronto-occipital Fasciculus (IFOF), Superior Fronto-occipital Fasciculus (SFOF), Superior Longitudinal Fasciculus (SLF), Anterior Corona Radiata (ACR), Posterior Corona Radiata (PCR), Superior Corona Radiata (SCR), Anterior Limb of Internal Capsule (ALIC), Posterior Limb of Internal Capsule (PLIC) and Retrolenticular part of the Internal Capsule (RIC). The overall results of this analysis are summarized in Table 3. Below, we provide more details about the results for each diffusion model. We also provide mean and standard deviation values for both TDC and CHD neonates for each of the diffusion measures in (Table 4 for TDC, and Table 5 for CHD).

**Table 3:**
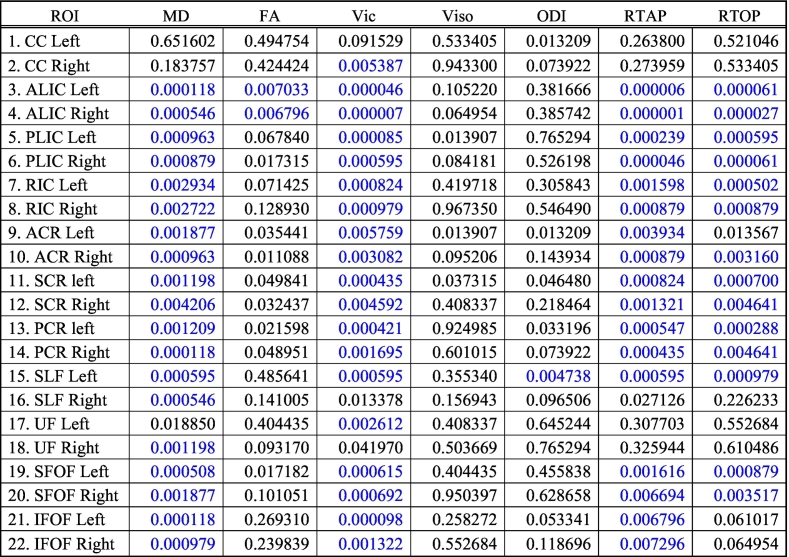
Correlation of diffusion measures with age in TDC subjects in all 22 white matter regions. Statistically significant results are colored in blue after FDR based multiple comparison correction.

**Table 4:** Mean and standard deviation values for each ROI across measures for typically developing control neonates (N = 16); R = right; L = left; *xE − 3, **xE + 3.

TDC neonates	MD*		FA*		Vic*		Viso*		ODI*		RTAP**		RTOP**	
	*xE − 3										**xE + 3			
ROI	Mean	Sd	Mean	Sd	Mean	Sd	Mean	Sd	Mean	Sd	Mean	Sd	Mean	Sd
1. L CC	1.5	0.08	365.0	19.3	259.0	27.1	194	44.6	104.0	9.6	2.0	0.14	77.5	7.0
2. R CC	1.5	0.07	362.0	20.7	276.0	22.3	216	39.8	109.0	9.1	2.0	0.15	77.3	7.8
3. L ALIC	1.1	0.06	263.0	17.2	221.0	23.8	8.5	5.4	221.0	15.3	2.2	0.20	100.0	11.3
4. R ALIC	1.2	0.06	270 0.0	20.4	220.0	24.8	13.5	13.2	213.0	9.2	2.2	0.19	98.9	9.6
5. L PLIC	1.0	0.04	412.0	19.4	295.0	18.5	13.5	10.4	146.0	10.9	3.0	0.27	141.0	13.4
6. R PLIC	1.0	0.04	409.0	24.3	289.0	19.6	18.4	14.1	144.0	10.6	3.0	0.28	141.0	13.8
7. L RIC	1.2	0.06	344.0	19.9	225.0	18.8	8.5	11.1	147.0	11.4	2.3	0.21	103.0	10.3
8. R RIC	1.2	0.05	339.0	21.9	215.0	18.0	9.8	9.0	139.0	14.5	2.2	0.21	101.0	10.6
9. L ACR	1.4	0.11	203.0	20.9	122.0	28.3	28.7	23.1	165.0	22.2	1.4	0.18	58.5	11.1
10. R ACR	1.4	0.12	205.0	22.6	123.0	33.6	33.1	37.2	156.0	30.3	1.4	0.21	57.9	13.2
11. L SCR	1.3	0.10	230 0.0	24.1	153.0	28.1	16.8	17.3	164.0	18.0	1.6	0.20	73.3	12.0
12. R SCR	1.3	0.09	235.0	24.8	153.0	25.8	16.6	18.2	158.0	21.7	1.6	0.19	72.9	11.0
13. L PCR	1.2	0.08	284.0	24.1	183.0	27.5	14.2	12.3	149.0	10.0	1.9	0.23	85.1	12.5
14. R PCR	1.3	0.07	279.0	26.3	174.0	26.8	16.1	9.8	144.0	18.0	1.8	0.22	81.2	12.5
15. L SLF	1.4	0.11	206.0	16.0	120.0	30.9	16.6	13.6	163.0	44.2	1.3	0.16	54.9	9.8
16. R SLF	1.4	0.08	189.0	19.7	120.0	24.2	8.0	7.4	188.0	28.5	1.3	0.13	54.2	8.3
17. L UF	1.2	0.05	257.0	21.3	185.0	18.6	6.6	10.3	162.0	21.9	1.7	0.13	78.2	9.3
18. R UF	1.2	0.05	260 0.0	16.9	187.0	21.7	10.6	21.0	161.0	21.2	1.7	0.12	76.0	6.9
19. L SFOF	1.3	0.09	234 0.0	25.5	172.0	27.9	14.8	14.6	181.0	24.1	1.8	0.22	80.0	12.9
20. R SFOF	1.3	0.08	228.0	28.6	174.0	24.5	17.9	20.8	190.0	26.0	1.7	0.21	79.6	12.1
21. L IFOF	1.2	0.06	280.0	16.1	195.0	21.3	6.7	4.0	170.0	20.8	1.9	0.10	82.1	6.5
22. R IFOF	1.2	0.06	278 0.0	24.6	186.0	19.8	8.6	7.1	161.0	22.3	1.8	0.14	79.8	8.0

**Table 5:** Mean and standard deviation values for each ROI across measures for congenital heart disease neonates (N = 19); R = right; L = left; *xE − 3, **xE + 3.

CHD neonates	MD*		FA*		Vic*		Viso*		ODI*		RTAP**		RTOP**	
	*xE − 3										**xE + 3			
ROI	Mean	Sd	Mean	Sd	Mean	Sd	Mean	Sd	Mean	Sd	Mean	Sd	Mean	Sd
1. L CC	1.6	0.09	330.8	25.8	230.0	31.6	216.0	51.5	114.0	18.0	1.7	0.18	62.5	9.1
2. R CC	1.6	0.07	329.2	24.4	243.0	31.2	235.0	45.6	117.0	15.3	1.7	0.18	62.5	8.8
3. L ALIC	1.2	0.05	236.3	21.0	187.0	22.7	10.4	12	215.0	15.0	1.9	0.19	81.5	11.3
4. R ALIC	1.2	0.05	241.9	21.9	182.0	23.6	10.3	11.6	205.0	14.4	1.9	0.21	80.9	11.9
5. L PLIC	1.1	0.04	383.7	24.3	260.0	21.0	4.8	5.5	141.0	14.0	2.7	0.24	122.0	12.7
6. R PLIC	1.1	0.04	388.0	21.1	256.0	19.8	7.5	6.3	139.0	12.6	2.7	0.25	122.0	13.1
7. L RIC	1.2	0.05	312.0	20.2	199.0	21.8	3.2	3.0	153.0	15.2	2.0	0.19	89.0	11.9
8. R RIC	1.2	0.06	308.3	18.8	190.0	21.8	7.7	10.0	143.0	21.2	2.0	0.19	87.0	11.5
9. L ACR	1.5	0.09	164.5	23.5	81.0	20.8	57.5	52.8	177.0	37.2	1.1	0.12	41.7	7.6
10. R ACR	1.5	0.10	164.4	27.7	77.2	24.8	60.1	53.9	173.0	53.5	1.1	0.14	40.9	8.5
11. L SCR	1.4	0.09	202.9	25.0	111.0	25.7	31.0	38.3	156.0	23.0	1.3	0.18	54.4	10.9
12. R SCR	1.4	0.09	207.6	22.9	115.0	25.3	29.0	35.8	148.0	21.3	1.4	0.17	56.4	10.9
13. L PCR	1.3	0.08	250.6	26.5	145.0	31.7	25.9	18	142.0	23.7	1.6	0.21	67.4	12.6
14. R PCR	1.4	0.07	250.7	18.3	135.0	21.4	32.2	26.6	134.0	17.5	1.5	0.14	63.1	9.0
15. L SLF	1.5	0.07	181.8	16.6	81.7	18.9	27.0	29.6	170.0	45.3	1.1	0.10	42.3	6.5
16. R SLF	1.5	0.08	163.7	16.7	88.0	20.1	22.5	27.4	198.0	43.3	1.1	0.11	44.5	7.0
17. L UF	1.3	0.07	222.8	26.1	162.0	22.5	25.0	27.2	168.0	26.4	1.5	0.11	62.9	7.4
18. R UF	1.3	0.05	213.6	18.9	156.0	17.3	10.7	18.9	179.0	22.7	1.5	0.14	64.7	8.9
19. L SFOF	1.4	0.09	195.6	24.4	123.0	28.8	38.6	39.8	171.0	20.0	1.4	0.18	56.6	11.4
20. R SFOF	1.4	0.07	190.1	26.8	123.0	22.1	26.0	40.5	172.0	22.3	1.4	0.15	57.3	9.8
21. L IFOF	1.2	0.05	256.5	18.1	165.0	17.4	6.0	4.3	159.0	18.6	1.6	0.12	68.6	7.4
22. R IFOF	1.3	0.05	260.5	17.7	161.0	19.0	4.5	5.2	145.0	19.7	1.6	0.14	68.5	8.7

#### 3.1.1 Diffusion Tensor Imaging (DTI)

##### 3.1.1.1 MD

**Fig. 1:**
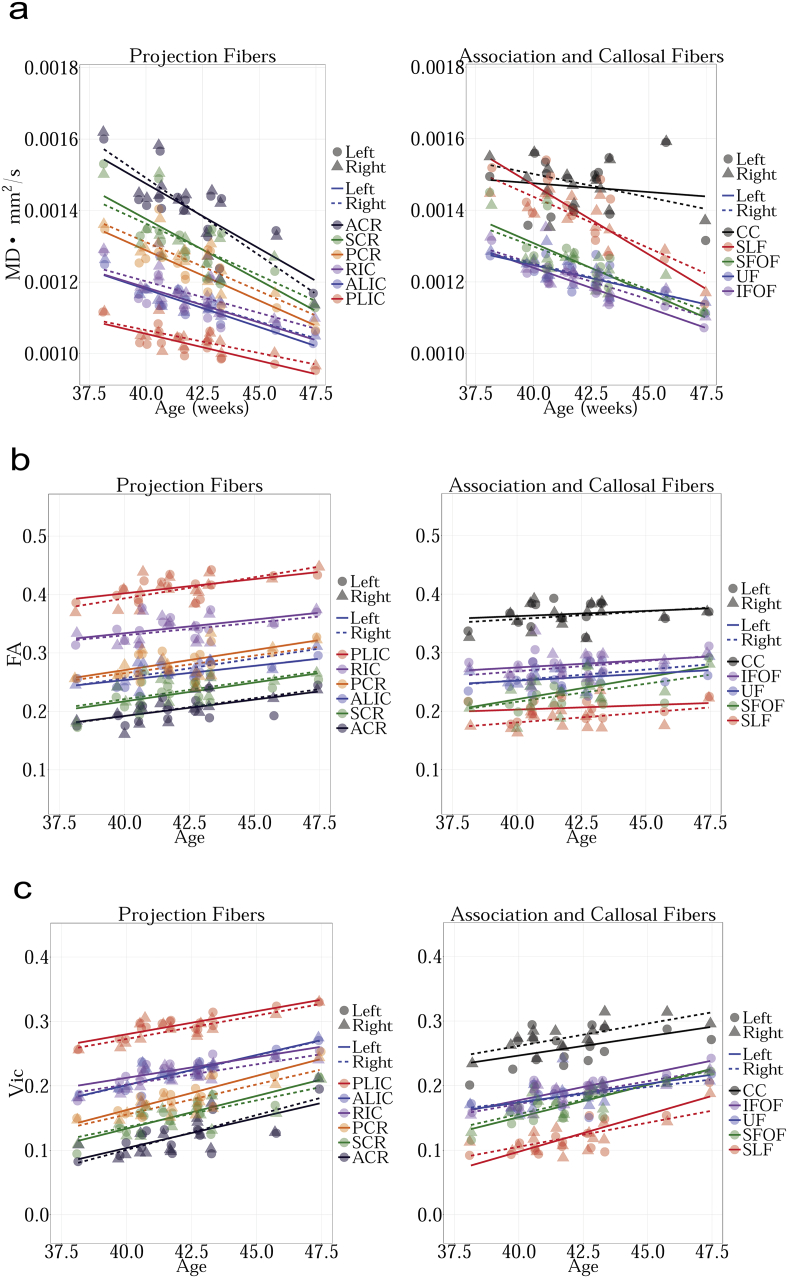

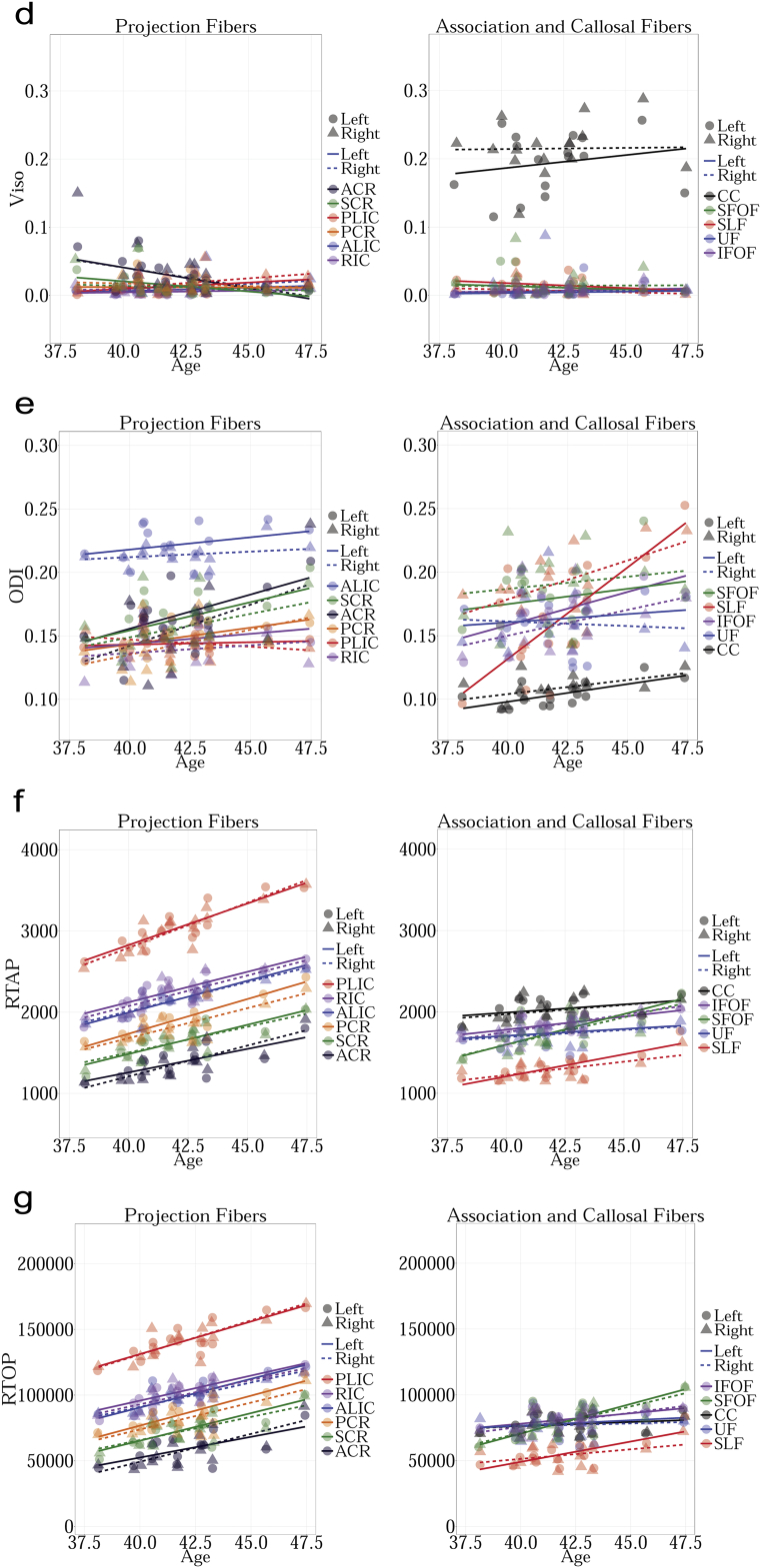
a. Regression analysis of MD with postnatal age in TDC neonates shows decreasing MD for bilateral ALIC, PLIC, RIC, ACR, SCR, PCR, SLF, SFOF, IFOF and right UF (FDR corrected p < 0.01). Other decreases were not significant. MD measures the amount of water diffusion in the brain tissue. b. Regression analysis of FA with postnatal age in TDC neonates shows increasing FA for bilateral ALIC (FDR corrected p < 0.01). Other increases were not significant. FA measures the relative diffusivity of water molecules along the WM fibers compared to the perpendicular or radial direction. c. Regression analysis of V_ic_ with postnatal age in TDC neonates shows increasing V_ic_ for bilateral ALIC, PLIC, RIC, ACR, SCR, PCR, SFOF, IFOF, right CC, left SLF and left UF (FDR corrected p < 0.01). Other increases were not significant. V_ic_ is derived from the NODDI model, and measures the intracellular volume fraction. d. Regression analysis of V_iso_ with postnatal age in TDC neonates shows no significant changes with age. V_iso_ is derived from the NODDI model, and measures the isotropic volume fraction. e. Regression analysis of ODI with postnatal age in TDC neonates shows increasing ODI for left SLF (FDR corrected p < 0.01). The remaining changes were not significant. ODI is derived from the NODDI model, and measures the fiber orientation dispersion index. f. Regression analysis of RTAP with postnatal age in TDC neonates shows increasing RTAP. Statistical significances were seen bilaterally in ALIC, PLIC, RIC, ACR, SCR, PCR, SFOF, IFOF, and left SLF (FDR corrected p < 0.01). The remaining increases were not significant. RTAP is derived from the Gaussian Mixture Model, and measures the return to axis probability. g. Regression analysis of RTOP with postnatal age in TDC neonates shows increasing RTOP. Statistically significant increases with age were seen bilaterally for ALIC, PLIC, RIC, SCR, PCR, SFOF and on the right for ACR and left for SLF (FDR corrected p < 0.01). The remaining increases were not significant. RTOP is derived from the Gaussian Mixture Model, and measures the return to origin probability.

MD measures the total amount of water diffusion in the brain tissue. Fig. 1.a shows changes in MD with age in TDC. The results can be summarized as follows:

1) CC, SLF, and ACR have the highest MD values.

2) PLIC has the lowest MD values.

##### 3.1.1.2 FA

FA measures the relative diffusivity of water molecules along the WM regions compared to the perpendicular or radial direction. Fig. 1.b shows cross-sectional age-related changes in FA in TDC. The results can be summarized as follows:

1) PLIC has the highest FA values.

2) SLF and ACR had the lowest FA values.

#### 3.1.2 Neurite Orientation Dispersion and Density Imaging (NODDI)

##### 3.1.2.1 V_ic_

V_ic_ is the intra-cellular volume fraction obtained through the NODDI model. Fig. 1.c shows changes in V_ic_ as a function of age in TDC. The results can be summarized as follows:

1) PLIC has the highest V_ic_ value.

2) SLF and ACR has the lowest V_ic_ value.

##### 3.1.2.2 V_iso_

V_iso_ measures the isotropic diffusion compartment in the tissue. Fig. 1.d shows changes in V_iso_ at different ages in TDC neonates. The results can be summarized as follows:

1) CC has the highest V_iso_ values.

2) RIC and IFOF have the lowest V_iso_ values.

##### 3.1.2.3 ODI

ODI models the orientation dispersion of fibers in tissue. Fig. 1.e shows age-related changes in ODI in TDC neonates. The results can be summarized as follows:

1. ALIC has the highest ODI values.

2. CC has the lowest ODI values.

#### 3.1.3 Gaussian Mixture Model (GMM)

##### 3.1.3.1 RTAP

RTAP is sensitive to the axonal density, diameter, and myelination. Fig. 1.f shows changes in RTAP as a function of age in TDC neonates. The results can be summarized as follows:

1. PLIC has the highest RTAP values.

2. SLF and ACR have the lowest RTAP values.

##### 3.1.3.2 RTOP

RTOP is thought to reflect the packing density and volume of fiber bundles (Fick et al., 2016). Fig. 1.g shows changes in RTOP with age in TDC. The results can be summarized as follows:

1) PLIC has the highest RTOP values.

2) SLF has the lowest RTOP values.

**Fig. 2:**
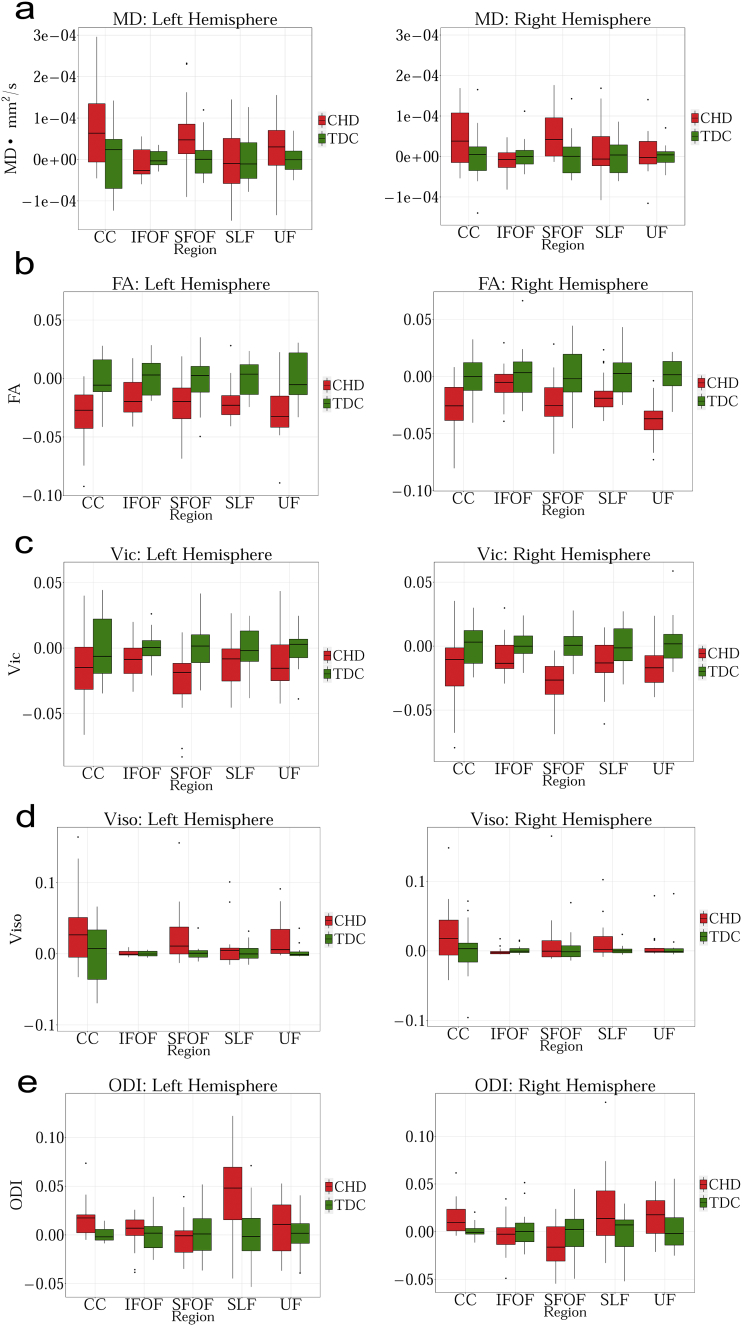

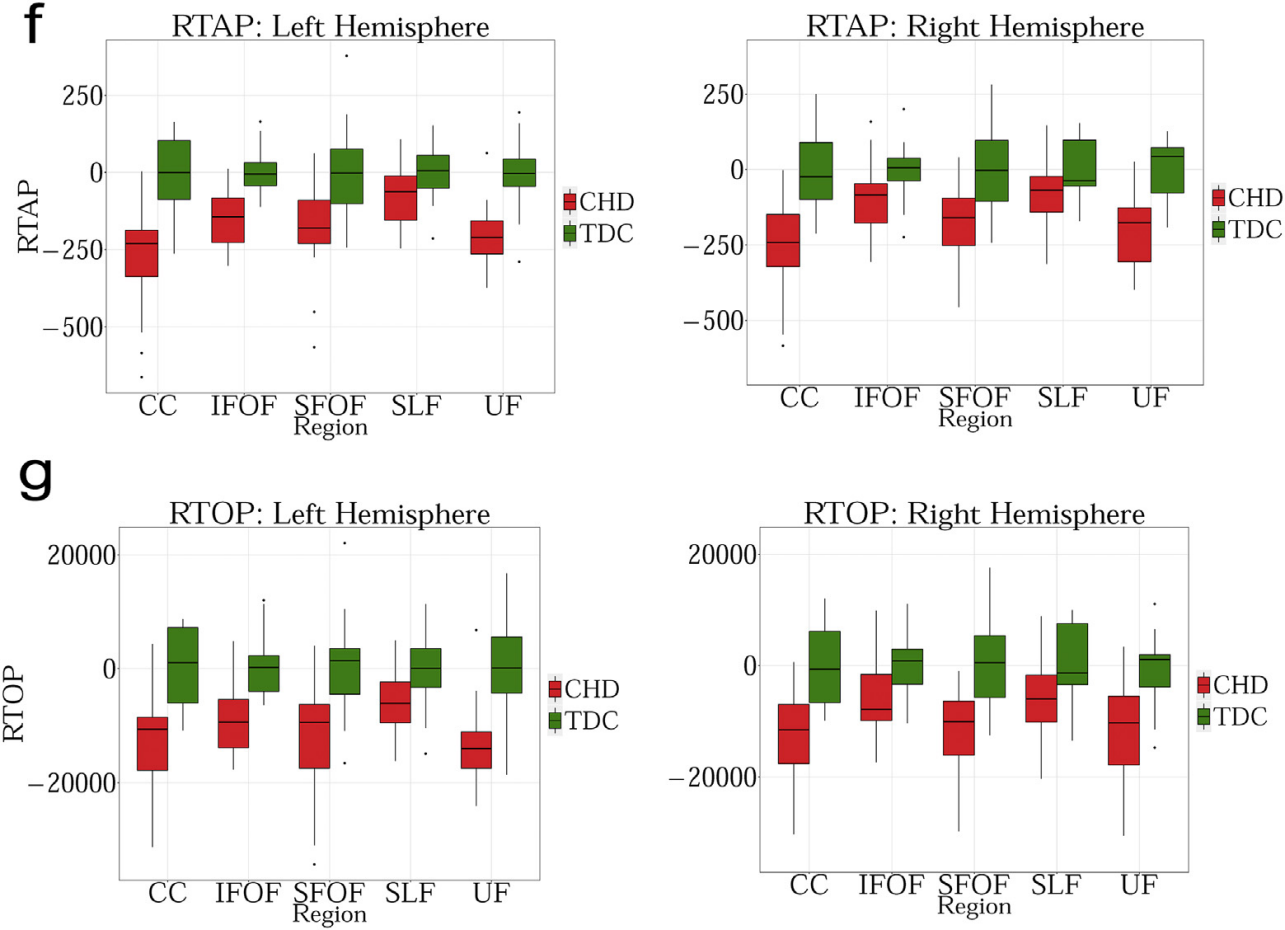
a. Group comparison for MD between CHD and TDC neonates in Corpus Callosum (CC), Inferior Fronto-occipital Fasciculus (IFOF), Superior Fronto-occipital Fasciculus (SFOF), Superior Longitudinal Fasciculus (SLF), Uncinate Fasciculus (UF). No statistical significant differences are observed in any of the tracts. Graph displaying statistical data based on the minimum, first quartile, median, third quartile, and maximum. b. Group comparison for FA between CHD and TDC neonates in Corpus Callosum (CC), Inferior Fronto-occipital Fasciculus (IFOF), Superior Fronto-occipital Fasciculus (SFOF), Superior Longitudinal Fasciculus (SLF), Uncinate Fasciculus (UF). FA is significantly lower for CHD in bilateral CC, UF and left SLF (FDR corrected p < 0.01). Graph displaying statistical data based on the minimum, first quartile, median, third quartile, and maximum. c. Group comparison for V_ic_ between CHD and TDC neonates in Corpus Callosum (CC), Inferior Fronto-occipital Fasciculus (IFOF), Superior Fronto-occipital Fasciculus (SFOF), Superior Longitudinal Fasciculus (SLF), Uncinate Fasciculus (UF). V_ic_ is significantly lower for CHD in bilateral SFOF (FDR corrected p < 0.01) Graph displaying statistical data based on the minimum, first quartile, median, third quartile, and maximum. d. Group comparison for V_iso_ between CHD and TDC neonates in Corpus Callosum (CC), Inferior Fronto-occipital Fasciculus (IFOF), Superior Fronto-occipital Fasciculus (SFOF), Superior Longitudinal Fasciculus (SLF), Uncinate Fasciculus (UF). No statistical significance is observed in any region. Graph displaying statistical data based on the minimum, first quartile, median, third quartile, and maximum. e. Group comparison for ODI between CHD and TDC neonates in Corpus Callosum (CC), Inferior Fronto-occipital Fasciculus (IFOF), Superior Fronto-occipital Fasciculus (SFOF), Superior Longitudinal Fasciculus (SLF), Uncinate Fasciculus (UF). ODI is significantly higher for CHD in the left CC (FDR corrected p < 0.01). Graph displaying statistical data based on the minimum, first quartile, median, third quartile, and maximum. f. Group comparison for RTAP between CHD and TDC neonates in Corpus Callosum (CC), Inferior Fronto-occipital Fasciculus (IFOF), Superior Fronto-occipital Fasciculus (SFOF), Superior Longitudinal Fasciculus (SLF) and Uncinate Fasciculus (UF). RTAP is significantly lower for CHD in the bilateral CC, UF, SFOF and left IFOF (FDR corrected p < 0.01). Note the improved separation of CHD from TDC with this measure. Graph displaying statistical data based on the minimum, first quartile, median, third quartile, and maximum. g. Group comparison for RTOP between CHD and TDC neonates in Corpus Callosum (CC), Inferior Fronto-occipital Fasciculus (IFOF), Superior Fronto-occipital Fasciculus (SFOF), Superior Longitudinal Fasciculus (SLF), Uncinate Fasciculus (UF). RTOP is significantly lower for CHD in the bilateral CC, UF, SFOF and left IFOF (FDR corrected p < 0.01). Graph displaying statistical data based on the minimum, first quartile, median, third quartile, and maximum.

**Fig. A.1:**
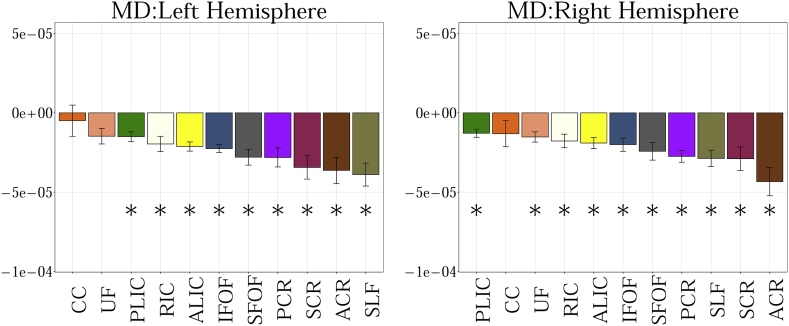
Showing the slope obtained from the linear fit between 11 regions of interest for MD. Statistical significance after FDR correction is shown with an asterisk (p < 0.01).

**Fig. A.2:**
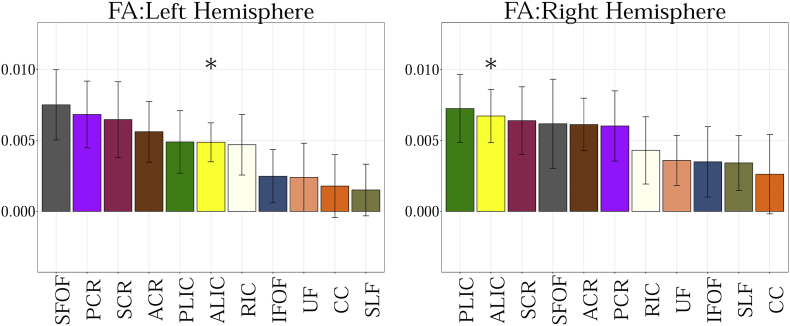
Showing the slope obtained from the linear fit between 11 regions of interest for FA. Statistical significance after FDR correction is shown with an asterisk (p < 0.01).

**Fig. A.3:**
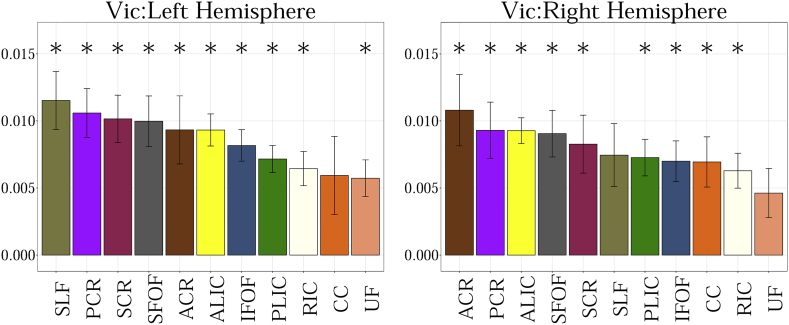
Showing the slope obtained from the linear fit between 11 regions of interest for V_ic_. Statistical significance after FDR correction is shown with an asterisk (p < 0.01).

**Fig. A.4:**
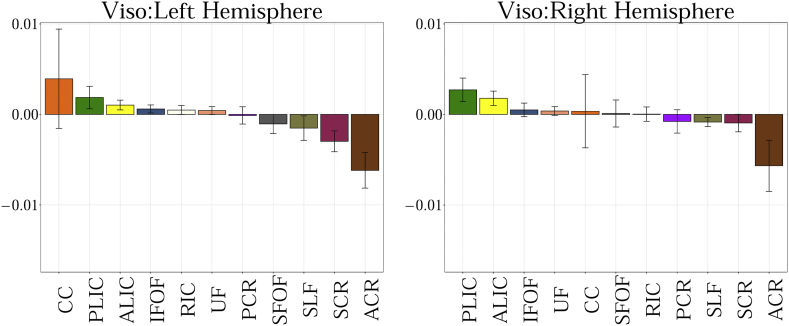
Showing the slope obtained from the linear fit between 11 regions of interest for V_iso_.

**Fig. A.5:**
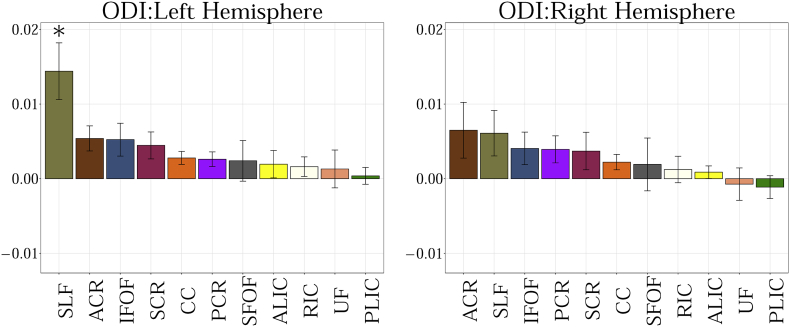
Showing the slope obtained from the linear fit between 11 regions of interest for ODI. Statistical significance after FDR correction is shown with an asterisk (p < 0.01).

**Fig. A.6:**
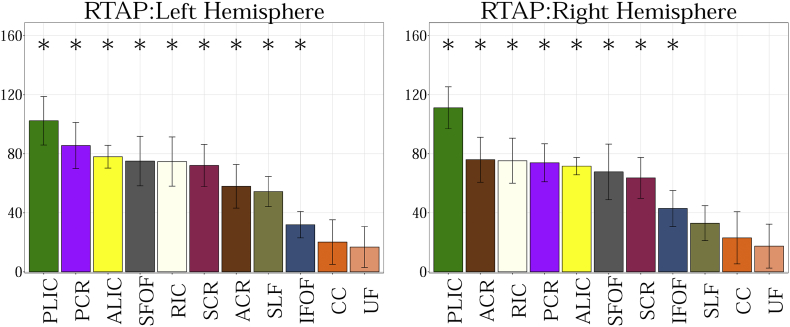
Showing the slope obtained from the linear fit between 11 regions of interest for RTAP. Statistical significance after FDR correction is shown with an asterisk (p < 0.01).

**Fig. A.7:**
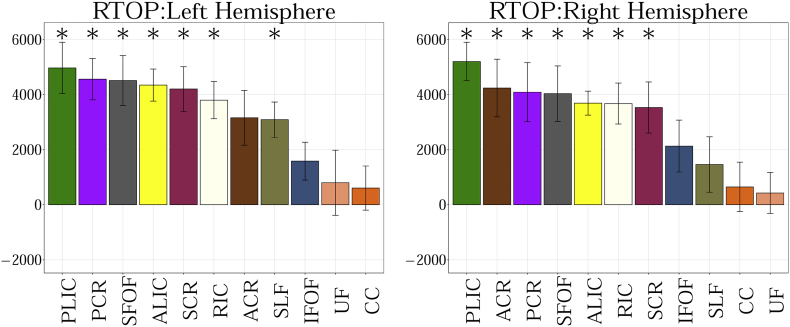
Showing the slope obtained from the linear fit between 11 regions of interest for RTOP. Statistical significance after FDR correction is shown with an asterisk (p < 0.01).

In supplement (Fig. A.1, Fig. A.2, Fig. A.3, Fig. A.4, Fig. A.5, Fig. A.6, Fig. A.7), we present the results from Fig. 2 in a different format to appreciate the slope (increase or decrease) of diffusion measures in different WM regions between TDC neonates at different ages. In particular, we ordered the slope of each region (for each measure) in decreasing order to fully understand the differential WM maturation between TDCs at different ages.

### 3.2 White matter regional abnormalities in CHD

We compared each of the diffusion measures between the TDCs and CHD subjects. An overall table of p-values for all measures is given in (Table 6), where we have reported the adjusted p-values after FDR-based multiple comparisons.

**Table 6:**
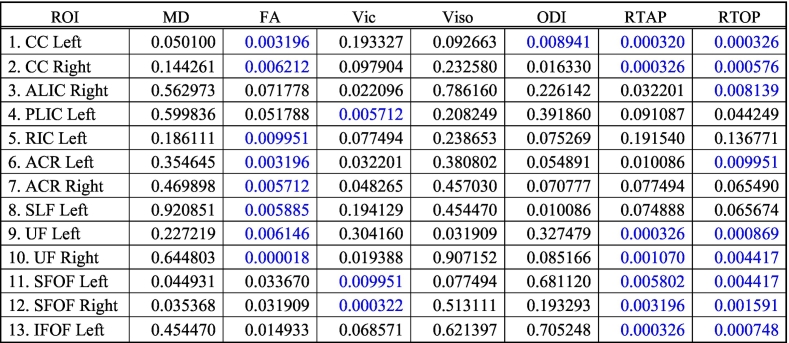
P values after correcting for multiple comparisons for ROIs. Blue color indicates statistical significance in group comparison between TDC and neonates with CHD (FDR corrected p < 0.01).

MD did not show any significant difference between TDC and neonates with CHD (Fig. 2.a). FA was statistically lower in CHD compared to TDC neonates in bilateral CC, ACR, UF, left SLF and left RIC (Fig. 2.b). V_ic_ was lower in the CHD subjects in the bilateral SFOF and left PLIC (Fig. 2.c). Orientation Dispersion Index (ODI) was statistically higher in CHD neonates in the left CC (Fig. 2.d), while V_iso_ showed no statistically significant differences for any WM structure (Fig. 2.e). RTAP was significantly lower in CHD in bilateral CC, UF, SFOF, and left IFOF (Fig. 2.f), while RTOP was statistically lower in CHD in several regions: bilateral CC, UF, SFOF, left IFOF, right ALIC and left ACR (Fig. 2.g).

Table 6 shows that measures derived from GMM detected more regional differences in white matter structures between CHD and TDC groups with more regions found to be statistically different using this model compared to the NODDI model. Further, the Cohen's d reported in Table 7, shows that the magnitude of effect sizes is larger for the GMM compared to the NODDI and DTI models.

**Table 7:**
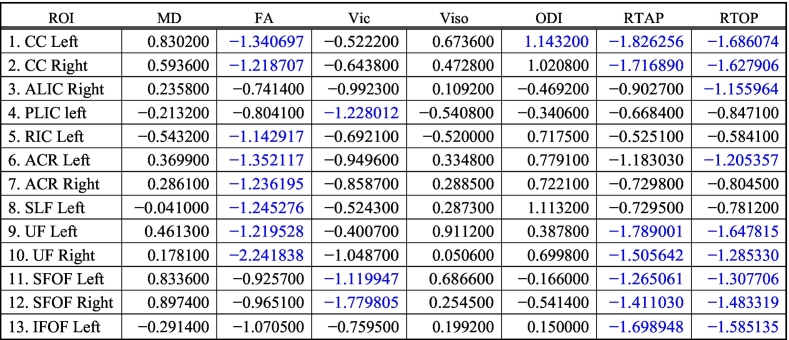
Cohen's d score reflecting the effect size magnitude. |d| < 0.2 “negligible”, |d| < 0.5 “small”, |d| < 0.8 “medium”, |d| > 0.8 “large”. Blue color indicates effect size for statistically significant ROIs.

## 4 Discussion

One of the key goals of our study was to characterize regional WM microstructure in typically developing neonatal brains using new advancements in dMRI. Thus far, past studies had used DTI, CHARMED-light and NODDI measures (Kunz et al., 2014; Qiu et al., 2015). In this study, we not only characterized regional WM structures with DTI measures (FA, MD) and NODDI measures (Vic, Viso, ODI), but we also used tissue model-free GMM measures (RTAP, RTOP). This allowed us to compare the sensitivity of different diffusion methods to study specific microstructural changes in the early postnatal weeks.

### 4.1 Developmental trajectories in TDC

#### 4.1.1 Projection fibers

The results obtained from DTI measures demonstrate changes in the WM regions between TDC neonates at different postnatal weeks. More specifically, an increase in FA and a decrease in MD are observed across all WM regions with an increase of age between TDC neonates. Furthermore, the PLIC, where partially mature myelin can be observed at birth (Brody et al., 1987), shows the highest value for FA and V_ic_ versus the lowest value for MD compared to other projection, association, and callosal fibers throughout the age range in our study. The results from RTOP and RTAP showed similar developmental trends in PLIC as the FA derived from DTI. The presence of myelination in PLIC decreases the extra-cellular space, making diffusion of water more restricted and hindered. Restriction of water with developing myelin increases the anisotropy of WM regions (Neil et al., 1998), leading to higher FA, V_ic_, RTOP and RTAP values in PLIC. Known trends in maturation from dorsal to ventral (Kinney et al., 1988) were also observed in MD, FA, V_ic_, RTAP, and RTOP.

#### 4.1.2 Corpus callosum

The results from our study show that CC, unmyelinated at birth (Kinney et al., 1988), has lower FA and V_ic_ values than PLIC but higher values than the other WM regions. Similarly, we observed the lowest ODI values in the CC compared to all other WM regions, which reflect high compactness and less dispersion of fibers. While the CC has high directional coherence of fibers, the partial volume from the ventricles might contribute to the lower FA compared to PLIC. We observed the highest isotropic volume fraction, V_iso_, in the CC, indicating the existence of partial volume effects.

Moreover, the results from RTOP and RTAP showed much lower values in the CC compared to PLIC. We note that both these measures are sensitive to the overall diffusional volume. Thus, due to the presence of myelinated axons in PLIC, the extracellular volume is lower and the contribution of restricted diffusional fraction higher, leading to higher RTOP/RTAP values. On the other hand, in CC, which has mostly unmyelinated axons at birth, the extracellular volume fraction is slightly higher, leading to lower RTOP and RTAP values. Another reason for lower values in CC might be due to partial volume with ventricles, which are in close proximity.

#### 4.1.3 Association fibers

We also observed differences in the pattern of maturation across different measures within association fibers of the ventral language pathway (UF and IFOF) showing greater maturation than those of the dorsal language pathway (SLF). Similarly, a past DTI study has shown that the SLF has a slow maturation rate extending into childhood (Zhang et al., 2007). This can also be observed in our results during the early postnatal weeks, as SLF has the lowest FA, V_ic_, RTOP, and RTAP values compared to other association fibers. Thus, our results show a heterogeneous maturation of different WM regions across the TDC population.

#### 4.1.4 Model comparison for development

We observed the expected pattern of change with age across the three models used. However, when comparing PLIC with CC using FA, we see much fewer differences between the values between these two WM regions as compared to the greater difference seen with GMM. This increase in FA could be either due to increase in myelination from the PLIC or coherence from the CC. The values obtained from V_ic_ were the closest between PLIC and CC, indicating that there might be a possibility of partial volume effects from the lateral ventricles in the CC affecting the DTI and GMM measures, and removing such effects in the NODDI model brought the values closer between these two regions (by modeling an isotropic diffusion fraction V_iso_).

However, it is also important to note that estimated parameter values for the NODDI model might be suboptimal as the diffusivity values that are by default fixed into the NODDI model may not be accurate for neonate's brains. For example, our estimate of the axial diffusivity in the TDC neonates in the CC is 0.0021 mm^2^/s, whereas the default value is 0.0017 mm^2^/s. Further, as shown in Jelescu et al. (2015), the estimation of the NODDI parameters might have several confounds, which could lead to over- or under-estimation of the volume fractions in neonatal data. We also note that fixing the diffusivity to the one found in the CC of TDC neonates also may not be optimal, as the axial diffusivity varies across different regions. In our study, the average axial diffusivity for SFOF was 0.0015 mm^2^/s, much lower than the axial diffusivity of 0.0021 mm^2^/s observed in the CC. This demonstrates that fixing axial diffusivity may be an inherent limitation of the NODDI model.

In summary, we found that all measures were sensitive to age-related changes in TDC neonates in the regional WM structure. However, DTI and NODDI models simplify the modeling of biological tissue thereby limiting its sensitivity to capture all the variations during the developmental period of the brain. Since GMM does not assume any tissue model, it allows more freedom to capture the variability of each WM region.

### 4.2 Differential development of WM in CHD

In this study, we identified region-specific WM abnormalities related to CHD. The differences between CHD and TDC were most prominent in the CC as well as the association fibers of the ventral language pathway (UF, SFOF, left IFOF) and dorsal language pathway (SLF). Furthermore, assessing WM regions with seven different dMRI measures, provided more information as to the nature of WM structural abnormalities in CHD neonates.

#### 4.2.1 Corpus callosum

The DTI results demonstrate the lower integrity of WM regions in CHD neonates. A previous DTI study in neonates with CHD demonstrated slower maturation of WM regions including the posterior part of the CC (Mulkey et al., 2014). Similarly, our result demonstrates lower FA in the CC among CHD neonates. Although FA is not specific to any particular type of biological change, lower FA might suggest that the compactness of CC is lower in CHD neonates. This also reflects the results obtained from ODI, as the ODI values in left CC for CHD neonates are significantly higher than in TDC neonates, suggesting greater dispersion of fiber bundles. Furthermore, we observe significantly lower RTOP and RTAP in the CC of CHD neonates. Since the corpus callosum in neonates primarily contains densely packed unmyelinated axons, lower RTOP and RTAP could be due to larger extra-axonal and extra-cellular volume, which implies fewer axons and cells in the CC of neonates with CHD. On the other hand, increased ODI implies incoherent axonal layout. These results demonstrate the possible factors contributing to the abnormalities seen in CHD, given the relationship between CC and several developmental disorders including developmental language delay (Fabbro et al., 2002; Paul, 2010).

#### 4.2.2 Ventral language pathway

In addition, the results from DTI demonstrate lower FA in the UF of CHD neonates implying delayed or abnormal maturity of this WM region. RTAP and RTOP were also significantly lower in this region. A previous DTI study in young adults with CHD showed lower FA in the left UF compared to controls, and the lower FA was positively correlated with verbal memory (Brewster et al., 2015). In our study, we were able to detect differences in FA, RTOP, and RTAP measures in the UF at birth among neonates with CHD. These results may indicate abnormal maturation pattern of UF during early postnatal weeks in CHD. Similarly, we observe statistically significant and lower RTOP and RTAP values in the left IFOF of neonates with CHD. We note that both the DTI and NODDI measures failed to detect these differences in the IFOF. Furthermore, UF and IFOF are believed to be part of the ventral language pathway involved in speech recognition, representation of lexical concepts and semantic processing of language (Chang et al., 2015). The ventral pathway is believed to be bilaterally organized within each hemisphere performing computationally different roles (Hickok and Poeppel, 2007). Although these roles are unclear, some studies have shown the dominance of right hemisphere for processing slow temporal features, whereas the left hemisphere supports rapid temporal features (Abrams et al., 2008). With increasing evidence of individuals with CHD facing neuro-developmental disorders including language dysfunctions, these results observed in the ventral pathway of language, with strong evidence of abnormalities in the left hemisphere, could be among the possible factors contributing to these impairments.

#### 4.2.3 Dorsal language pathway

Our results also demonstrate significantly lower FA in the left SLF of neonates with CHD. SLF is part of the dorsal language pathway which is responsible for the phonological processing of language (Chang et al., 2015). Unlike the ventral pathway, the dorsal pathway is strongly left hemisphere dominant (Hickok and Poeppel, 2007). The result in our study implies lower integrity of the left SLF in neonates with CHD, which could also contribute to language dysfunctions faced later in life. Although, we observed lower RTOP and RTAP in the SLF, there was no statistically significant difference after correction for multiple comparisons. It is important to note that the SLF is among the least mature WM regions observed during the first postnatal weeks, and has slower maturation and myelination rate than the regions of the ventral pathway in postnatal years (Zhang et al., 2007; Brauer et al., 2013). Given the slow maturation rate of SLF, the interpretation of abnormalities in early postnatal weeks for neonates with CHD is difficult.

#### 4.2.4 Model comparison

Measures derived from the NODDI model showed far fewer statistical differences than those from DTI and GMM measures, indicating a lower sensitivity of the NODDI measures in capturing differences between CHD and TDC. Further, GMM-derived measures showed the highest sensitivity in terms of the effect size differences observed between TDC and CHD population. By carefully changing the default parameters, the sensitivity of NODDI might improve, yet it still might be suboptimal for several regions as the axial diffusivity is regionally very different (as discussed earlier). Further, axial diffusivity also might differ substantially in developing population and those affected by diseases. These reasons make it difficult to optimize this parameter in the NODDI model. Hence, in our study, we used default parameters setting in the NODDI mode. Overall, our study leveraged advanced diffusion imaging to obtain several parameters which allowed us to observe region-specific WM maturation in TDC and detect abnormalities in neonates with CHD in early post-natal weeks.

#### 4.2.5 Limitation

Our study is limited in that 3 subjects were imaged postoperatively and 4 of 19 subjects (two imaged preoperatively and two imaged postoperatively) showed a recent injury on diffusion imaging. However, these acute lesions were small and, since recent, only two lesions were associated with decreased FA. Therefore, these lesions are likely to have little effect on our findings. Our study is also limited in assessing functional impairments associated with language development in the early postnatal weeks because speech production specific to native language only progresses between 6 months and the first year of the postnatal year (Dubois et al., 2016). Therefore, a future goal would be to perform a longitudinal study among CHD neonates, which in turn might help us understand whether the abnormalities associated with WM regions seen in early postnatal weeks might be associated with neurodevelopmental deficits including language development later during childhood and early adulthood. This might allow early interventions among neonates with CHD to help improve the cognitive outcomes associated with these WM regions because plasticity of the brain is very high during the early phase of development. Another limitation is that our groups were not gender-balanced, which may also contribute to differences between TDC and CHD populations.

## 5 Conclusion

Our results demonstrate the feasibility of using advanced multiple b-value diffusion imaging to characterize regional white matter maturation in neonatal brains and to detect micro-structural abnormalities associated with CHD. Our analysis of 22 different WM regions (both hemispheres) in TDC neonates showed significant changes in all diffusion metrics but MD and Viso in 12 different regions (i.e., FA, Vic, ODI, RTOP and RTAP changed with age). Thus, in this cross-sectional study, these 5 measures were sensitive to microstructural changes with age. Our analysis also showed statistically significant group differences between CHD neonates and TDC, with RTAP and RTOP providing the strongest statistical significance in terms of effect sizes and the number of abnormal regions. Furthermore, a major finding from our study indicates the presence of WM differences (lower axonal and cellular packing density and volume) in bilateral CC and UF and left IFOF and left SLF in CHD. To the best of our knowledge, this is the first study to use advanced diffusion measures to better characterize regional WM differences in congenital heart disease in a systems based approach. The left lateralization of these differences in CHD suggests that the delayed language development often observed in CHD may be connected to the abnormalities seen in our study in the early postnatal weeks. Further, this study also compared different models used in the diffusion MRI field and found that the GMM and DTI measures are more sensitive at detecting differences in tissue structure than the NODDI model, as measured in terms of the number of regions found to be abnormal. Long-term follow-up of the CHD neonates is planned to assess their cognitive outcomes especially in the domain of language to determine the predictive power of these differences. Moreover, our work may aid in the monitoring of future early interventions targeting functions related to these specific WM regions.

The following are the supplementary data related to this article.
